# Supplementary material for: A service provider perspective in Irish horseracing on the availability of retirement specific support services for professional jockeys and perceived barriers and facilitators to their use
Source: PLoS One. 2026 Apr 30;21(4):e0348055. doi: 10.1371/journal.pone.0348055 (PMC13132420; doi:10.1371/journal.pone.0348055)
Supplement: S1 Appendix — (DOCX) [file pone.0348055.s001.docx]

1. Can you tell me about your role with the industry?

● How long have you been in this current role?

2. Are you aware of any services/ supports/ education you provide to jockeys in preparation

for or after retirement?

● Is the service well received?

● Are there any problems with it in your opinion?

3. In relation to retirement do you think a service should be in place to aid jockeys with the

transition?

● Explain service (Psychological/financial planning/ career planning)

4. What is your opinion on the need for a jockey to prepare for retirement from a young

age/ at the start of their professional career?

● What do you think would be the benefits to the jockey?

● Can you think of any negatives with the idea of pre planning for retirement?

● Do you think this idea would be received well by stakeholders?

● How do you think stakeholders would feel about jockeys having a dual career?

5. Do you think the current services (if they exist) require change?

● What changes should be made?

● Do you have an ideas you think should be included in the development services

provided?

6. What changes should be made?

● What organisation should make these changes?

● Should there be cross organisation corroboration in this area?

● Do you think there is an appetite for these services?

7. What do you think the benefits of having a retirement structure and support service in

place for jockeys would be?

8. What are the barriers to (what stands in the way of) developing such a structure and

implementing it?

9. What would facilitate or encourage (help) change?

Probing questions:

1. Can you expand more on that point?

2. Is there anything else you would like to add?

3. Can you think of what could be done differently in this situation?

4. Can you explain what you mean?
