# Supplementary material for: A service provider perspective in Irish horseracing on the availability of retirement specific support services for professional jockeys and perceived barriers and facilitators to their use
Source: PLoS One. 2026 Apr 30;21(4):e0348055. doi: 10.1371/journal.pone.0348055 (PMC13132420; doi:10.1371/journal.pone.0348055)
Supplement: Attachment — Submitted filename: PLOS One Rebuttal Letter.docx [file pone.0348055.s003.docx]

Dear Reviewers,

I would like to thank you sincerely for your valuable comments and suggestions on the manuscript. I have endeavoured to answer your comments and make changes accordingly. I hope that you feel the manuscript reads better now and is stronger as a result of the changes made. I am grateful that you feel the manuscript could be impactful in its area and I hope that it will be received well.

Please find the replies to your comments and the details of the changes made below.

Kind regards,

Laura Langton

| **Reviewer 1 Comments:**  Given that the Retired Jockeys Questionnaire (RJQ) is referenced in the study, have the authors considered turning this into a mixed-methods study by adapting the RJQ to the Irish context before carrying out the semi-structured interviews? | **Rebuttal:**  Thank you for this comment. While this would be interesting when studying the jockey cohort themselves we felt it important to firstly gain the thoughts and experiences of services providers within the industry. This is definitely something to think about when further exploring the area with jockeys. |
| --- | --- |
| Perhaps add a line or two to explain what the Big Q paradigm is so as not to confuse readers. | Thank you for this comment, it makes sense to expand a little more for the readers understanding. I have now added an extra sentence to explain the Big Q paradigm in more detail.  L. 125-129 |
| Reference for "A study of young Finnish athletes" (line 669) missing. | Thank you. I have added the reference both in text and in the reference list. L. 674 |
| There are a few grammatical errors here and there, but they do not hinder meaning. | Thank you, I have asked a member of the research team to re-read the manuscript and hopefully amendments have been made accordingly. |
| **Reviewer 2 Comments:**  Intro: 100 - 103 - this paragraph needs more explanation  107 - it is unclear whether this is direct quote? if so it needs to be cited appropriately  110 - there is a need to explain the rationale of why the focus of the study are industry stockholders and not speaking directly to jockeys themselves? this is a serious omission that needs to be addressed. It does get a mention appropriately in the last paragraph of the article, but needs to addressed in a more substantial way dealer in the article. | L 98-105 Some adjustments have been made to the wording of this paragraph. I hope that it is cleared now.  L. 107 This is not a direct quote. My language was difficult to decipher this. I have changed the wording from ‘states’ to ‘acknowledges’.  L. 114-117 Thanks you for this comment. It make a lot of sense to add this information earlier in the article, as well as at the conclusion. Information regarding future research on jockeys has been added to the introduction section. |
| methodology 158 - who is "the researcher" in this context? lead author? 159 - this sentence seems a bit vague "small number of experts" was the guide validated by all listed contributors in this article? or was it a sub-section of the group? 263 - check style guide "(dual career)" should this be [duel career]? | L. 162 “the research” has been replaced by “lead author”.  L. 162 Thank you. “Small number of experts” has been replaced. The interview guide was critiqued by the full research team (named authors).  Thank you. From the articles I have read and using the spell check I believe that dual career is the term commonly used. |
| Discussion: 565 - might better read - "as outlined in the introduction..."  676 - 689 - is the endorsement about the role of CMO or the personality of the current CEO? | L. 570 Thank you, this has now been changed to “As outlined in the introduction”.  Wording has been changed to ensure it is the role of the CMO, rather than the personality of the CMO:  “*Endorsement from the professional working as CMO..”* |
| **Editor Comments:**  Introduction: Line 58: Change word services to service as it is used in singular context within the sentence. Line 58 – 61: Why is the word “the” capitalised for each stakeholder – is it a formal title? It is confusing to read with so many The’s mid-sentence. If “The” is not in formal name of stakeholder, perhaps it might be better to have the Injured Jockeys Fund, the Racing Academy and Centre of Education, and the Jockeys Trust. Lines 95-96: Should there be a reference for the social benefits sentence?  Line 109: Similar to point raised above, should it be the Jockey Pathway rather than The Jockey Pathway? | Thank for your valuable comments.  L. 57 services changed to service  Thank you for this comment. There was no specific reason for the use of capital letters. I have changed where appropriate based on this feedback.  L. 95 Reference added.  L. 109 This has been changed to “the Jockey Pathway” |
| Methodology: Was this study protocol ever registered on any online databases? If so, state this in the methodology section.  Line 122: Clarify who “the researcher” is – lead author?  Line 124: Expand or define “the Big Q paradigm”.  Line 145: Ethical approval reference number should be stated here.  Line 159: How many experts, and what classified them as an expert? Perhaps reference the guide here too (Supporting Information).  Line 176: Zoom initially should have correct branding here – i.e. Zoom (Zoom Communications, Qumu Corporation, Minneapolis, Minnesota, Unites States).  Line 179: Avoid use of / in manuscript. Perhaps state “or indeed” instead. | No the protocol was never registered.  L. 125-129126 Researcher changed to “lead author”  Thank you for this comment, it makes sense to expand a little more for the readers understanding. I have now added an extra sentence to explain the Big Q paradigm in more detail.  L. 150 Thank you, reference number added.  Thanks you. The papers co-authors are the research team. This information has been added to the manuscript (L. 162-163).  This branding has been added (L. 180-181)  Thanks you. This suggested change has been made (L.184) |
| Results:  No real mention of the demographics of the stakeholders. I appreciate that this may be a small industry and confidentiality is important, but even a breakdown of sex, age, experience and role within industry would be insightful here.  Line 349: Should “highlighting” be “Highlighting” (Capital letter at start of subtheme?  Line 367: Similar to above, should “endorsement” be “Endorsement”? | Thank you for this comment. I have now added the breakdown of sex (L. 142). I have also added some information regarding the roles of those involved in the study (L.148-150):  *“Participants included those who actively provide support services to jockeys, those who organise the support service provision as well as those in management roles within their organisations.”*  L. 354 Capital letter added.  L. 372 “endorsement” changed to “Endorsement”. |
| Discussion: Agree with Reviewer 2, in that this section is very lengthy. I would suggest taking on board the suggestions of Reviewer 2 within the revision process.  Critical appraisal for the Horse Racing Industry | Thak you for this comment. I have reviewed the discussion and in line with the comments made by reviewer 2, I have removed the section regarding metal health. I think makes for a more coherent discussion section that is fully in line with the results of this study.  Reviewer 2 highlighted the need for critical appraisal regarding stigma created by the industry itself. I have added this to the conclusion section of the paper (L. 761-768). Please do let me know if this suffices:  *“The culture within the sport of Irish horseracing and insufficient cross organisation communication were the main perceived barriers by industry service providers identified to the use of support services. It is important for an individual working within the horse racing industry and those providing support services to be made aware of these barriers so that changes can be made to make the Irish jockey more likely to use these support services going forward.”* |
|  |  |
