# Supplementary material for: A service provider perspective in Irish horseracing on the availability of retirement specific support services for professional jockeys and perceived barriers and facilitators to their use
Source: PLoS One. 2026 Apr 30;21(4):e0348055. doi: 10.1371/journal.pone.0348055 (PMC13132420; doi:10.1371/journal.pone.0348055)
Supplement: Attachment — Submitted filename: An industry perspective in Irish horseracing.docx [file pone.0348055.s002.docx]

This qualitative study explores industry stakeholders' perspectives on retirement support services for Irish professional jockeys. Through interviews with 11 service providers, the authors identify barriers (industry culture, communication gaps) and facilitators (flexible education, career normalization, exemplar programs) to service uptake. While addressing an important gap, the study has a critical limitation: **it excludes the voices of jockeys themselves**, presenting only service providers' perceptions of what jockeys need.

**MAJOR CONCERNS**

**1. Fundamental Sample Limitation**

**Critical Issue:** The study only interviews service providers, not jockeys.

- This is like evaluating a restaurant by asking only the chefs, never the customers
- The title "An industry perspective" is misleading—it should explicitly state "service provider perspective"
- There's likely a significant gap between what providers *think* jockeys need and what jockeys *actually* experience
- The authors acknowledge this (lines 114-117, 767-770) but don't adequately justify why they didn't include both groups from the start

**Impact on Validity:** The findings represent assumptions rather than lived experiences. For example, when discussing "stigma toward help-seeking," we hear providers' interpretations, not jockeys' actual fears or concerns.

**2. Methodological Weaknesses**

**Sample & Data Collection:**

- Only 11 participants for an entire industry seems insufficient
- Interview duration varies widely (16-48 minutes)—suggests inconsistent data depth
- Inclusion criterion of "1 year in role" is arbitrary and inadequately justified
- Pilot interview conducted with someone who didn't meet inclusion criteria (why?)

**Analysis Rigor:**

- No evidence of inter-rater reliability or multiple coders
- Only "peer debriefing" mentioned for rigor—insufficient for qualitative research
- Unclear how many researchers were involved in coding
- Risk of confirmation bias given all authors are "insiders" who've worked with jockeys for years

**3. Insider Bias & Reflexivity**

**Concern:** All authors have extensive history working in this industry.

- While they acknowledge using a "Big Q" paradigm and insider status, there's minimal discussion of how they managed potential biases
- No reflexive journal mentioned
- No discussion of how their positions might have influenced what participants felt comfortable saying
- Given small community size, power dynamics between researchers and participants may have affected data

**4. Overly Long, Repetitive Discussion**

The discussion section (300+ lines) is approximately **30-40% too long**:

- Excessive repetition of findings
- Some sections (e.g., mental health) tangentially related to core findings
- Could be more focused and impactful if condensed
- Theory is mentioned but not deeply integrated—feels added rather than foundational

**5. Weak Practical Recommendations**

While suggestions sound good (CPD, flexible education, career mapping), they lack depth:

- **Who pays?** No discussion of funding or economic feasibility
- **How to change culture?** Culture change is notoriously difficult; suggestions are superficial
- **Trainer resistance?** Identified as major barrier but no concrete solutions offered
- **Implementation timeline?** No roadmap provided

The recommendations reflect service providers' ideals rather than pragmatic, evidence-based solutions co-created with jockeys.

**6. Theoretical Framework**

- Multiple theories mentioned (Athletic Identity, Self-Determination Theory, Psychological Safety) but loosely applied
- No unified theoretical lens guiding interpretation
- Theory feels "sprinkled in" rather than integral to analysis
- Would benefit from consistent application of one or two frameworks

**7. Ethics & Confidentiality**

- Irish horse racing is a small community—even anonymized, participants may be identifiable
- Quotes are detailed enough that insiders could recognize speakers
- No discussion of how this risk was managed or communicated to participants

**8. Missing Critical Voices**

**Trainers:** Portrayed as barriers to change, yet their perspectives are absent. This is problematic—you cannot fairly characterize a group without hearing from them.

**Jockeys:** As mentioned, their absence is the study's fatal flaw.

**9. Presentation Issues**

- Some grammatical errors (though meaning is clear)
- Inconsistent capitalization of stakeholder names (lines 58-61)
- Formatting of subthemes inconsistent (lines 349, 367)
- References occasionally missing (line 669—now corrected)

**10. Generalizability**

- Findings are highly context-specific to Irish horse racing
- Limited discussion of how cultural differences might affect transferability to UK, Australia, or other racing jurisdictions
- The "uniqueness" of jockey culture is asserted but not compared systematically to other sports
