# Supplementary material for: A service provider perspective in Irish horseracing on the availability of retirement specific support services for professional jockeys and perceived barriers and facilitators to their use
Source: PLoS One. 2026 Apr 30;21(4):e0348055. doi: 10.1371/journal.pone.0348055 (PMC13132420; doi:10.1371/journal.pone.0348055)
Supplement: Attachment — Submitted filename: Response to Reviewers_PLOS ONE.docx [file pone.0348055.s004.docx]

Dear Editor,

Thank you for the opportunity to revise and resubmit our manuscript. We are grateful to you and the reviewers for your constructive and insightful feedback, which has helped to significantly strengthen the quality and clarity of the paper.

We have carefully addressed all comments raised by the reviewers and have revised the manuscript accordingly. A detailed, point-by-point response outlining how each comment has been addressed is provided below. Revisions have also been clearly indicated within the manuscript.

We believe these revisions have improved the rigour, clarity, and overall contribution of the study, and we hope that the manuscript is now suitable for publication in your journal.

Thank you for your time and consideration. We look forward to your feedback.

Yours sincerely,

Laura Langton

**Reviewer 1 (Yellow Highlights on manuscript)**

| Comment | Response | Changes |
| --- | --- | --- |
| Please consider revising the title so it clearly reflects that the study is written from a service-provider perspective. | Thank you for your comment, I have made a change to the title to include the terminology ‘service providers’ | L. 1-2  A service provider perspective in Irish horseracing on the availability of retirement specific support services for professional jockeys and perceived barriers and facilitators to their use. |
| It would also strengthen the paper if the limitations section were expanded to openly acknowledge the absence of jockey voices, the risk of insider bias, the limited generalisability of the findings, and the potential for confirmation bias. | Thank you for your comment. A specific limitations section has been added to address these comments. | L. 755-766  Several limitations should be considered when interpreting the findings of this research. First, this study reflects the perspectives of service providers and does not include the voices of current or retired jockeys. While participants offered insight into the availability a supports, their perspectives may not fully capture how these services are perceived or experienced by the jockeys themselves. Future research incorporating jockey perspectives would provide a more comprehensive understanding of service accessibility and engagement. Second, because participants operate within the system responsible for delivering these supports, there is potential for insider bias that may shape how services and potential barriers, and facilitators are described. Third, the findings are situated within the specific organisational and cultural context of Irish horseracing, which may limit transferability to other racing jurisdictions or sporting contexts. Finally, while coding procedures were used to enhance analytic rigour, qualitative interpretation remains subject to potential confirmation bias. |
| To avoid the study feeling incomplete, it would be helpful to explicitly commit to Phase 2, outlining a clear plan—including timeline, funding considerations, and methodological approach—for interviewing jockeys. | Thank you for your comment. I have added this information to the conclusion section. | L. 782-785  These should be explored further by gaining insight into the jockeys’ views on this topic. The research team have committed to carrying out this qualitative research, and have the support of the IHRB to do so. Both current and retired jockeys will be interviews to explore their perspectives. |
| The discussion section could also be tightened by about 30%, removing repetition and trimming content that does not directly contribute to retirement support (including the mental-health section if it is not fully integrated). | Thank you for this comment. On reflection I can see how the mental health section does not deepen the current studies findings. I have now removed it. | Mental Health section removed from manuscript |
| Adding a reflexivity statement would meaningfully enhance the transparency of the work by explaining how your insider role shaped data collection and interpretation | Thank you for your comment. A reflexivity statement was added to the Rigor section in methodology. | L. 210-214  Consistent with reflexive thematic analysis, the researcher acknowledges their active role in generating meaning from the data. Familiarity with the horseracing context informed both engagement with participants and interpretation of their accounts. Reflexive practice was maintained throughout the analytic process, with ongoing consideration of how prior knowledge and assumptions shaped analytic decisions and theme development. |
| The practical recommendations would benefit from deeper detail as well, especially regarding funding, implementation barriers, strategies for engaging trainers, and realistic rollout timelines. | Thank you for your comments. I have given more detail to the section on practical recommendation to discuss these point in more detail. | L. 775-778  These should be explored further by gaining insight into the jockeys’ views on this topic. The research team have committed to carrying out this qualitative research and have the support of the IHRB to do so. Both current and retired jockeys will be interviews to explore their perspectives.  L.781-784  The involvement of the IHRB in this research highlights the willingness on their behalf to implement recommendation made. Further research is needed to explore potential barriers that may arise on use of support services based on both jockey and trainer perspectives. |
| You may want to clarify your theoretical framework by using one or two consistent lenses (or explicitly noting the exploratory nature of the study) | Thank you for this comment. I have reworded the aim of the study in the introduction section to explicitly note the exploratory nature of the study. | L. 114-117  The aim of this research is to firstly explore the thoughts of the stakeholders who are providing these support services with the intention that further research can then explore the thoughts of both current and retired jockeys on this topic, to gain an even deeper understanding and alternative perspective. |
|  |  |  |
| Reflect on power dynamics—specifically how your organisational relationships may have influenced what participants felt comfortable sharing. | Thank you for your comment. A section on potential power dynamics has been added to the rigor section. | L. 215-223  Given the small and interconnected nature of the Irish horseracing industry, potential power dynamics between the researcher and participants should be acknowledged. The researcher’s familiarity with the sector may have influenced how comfortable service providers felt discussing limitations in current support provision or barriers affecting jockeys’ engagement with services. Participants may have moderated their responses to protect organisational relationships or reputations within the industry. To reduce this influence, confidentiality and anonymity were emphasised, and participants were informed that the research was independent of governing bodies; however, this potential influence should be considered when interpreting the findings. |

**Reviewer 2 – Blue highlights on manuscript**

| There's a "2" in "support 2 services" that needs removing. Also, the title is quite long - try to get it under 15 words. | Thank you for the comment – I have re-worded. The title and hopefully it is more concise and clearer as a result | L. 1-2  A service provider perspective in Irish horseracing on the availability of retirement specific support services for professional jockeys and perceived barriers and facilitators to their use. |
| --- | --- | --- |
| No limitations section: This is a significant omission. Every research paper needs to discuss its limitations. Please add a paragraph addressing things like: only talking to stakeholders (not jockeys themselves), the small sample size, potential bias from being insiders in jockey research, and that findings come from just one country. | Thank you for your comment. I have added a limitations section now to address both reviewers comments. | L. 757-768  Several limitations should be considered when interpreting the findings of this research. First, this study reflects the perspectives of service providers and does not include the voices of current or retired jockeys. While participants offered insight into the availability of support services, their perspectives may not fully capture how these services are perceived or experienced by the jockeys themselves. Future research incorporating jockey perspectives would provide a more comprehensive understanding of service accessibility and engagement. Second, because participants operate within the system responsible for delivering these supports, there is potential for insider bias that may shape how services and potential barriers, and facilitators are described. Third, the findings are situated within the specific organisational and cultural context of Irish horseracing, which may limit transferability to other racing jurisdictions or sporting contexts. Finally, while coding procedures were used to enhance analytic rigour, qualitative interpretation remains subject to potential confirmation bias. |
| Abstract: Your abstract is vague about what you actually found. Instead of saying "themes were identified," tell us what those themes were and give us some numbers. | Thank you for your comment. There is now a section in the abstract detailing the themes. | L. 25-29  The higher order themes identified as barriers to use of transition support services, were the culture within the Irish horseracing industry, and insufficient cross organisation communication. Higher order themes relating to facilitators of engagement were accessible further education opportunities for jockeys, normalising career planning and the development of an exemplar support service for jockey career transitions**.** |
| Research questions not explicit: Right now you say you want to "explore perceptions" which is quite general. At the end of your introduction, add clear numbered research questions like: o RQ1: What support services currently exist for Irish jockeys? o RQ2: What prevents jockeys from using these services? o RQ3: What would help jockeys use support services more? | Thank you for your comment. I have now added a more concise and definitive aim and followed it with detailed RQ’s. | L. 111-120  The aim of this study was to explore the perceptions of key stakeholders involved in the delivery of support services within Irish horseracing regarding the current provision of career and retirement support for jockeys. Specifically, the study sought to address the following research questions:  RQ1: What support services are currently available to assist Irish jockeys in preparing for retirement from professional horse racing?  RQ2: What barriers do stakeholders perceive as limiting Irish jockeys’ engagement with these support services?  RQ3: What facilitators do stakeholders perceive as promoting Irish jockeys’ engagement with support services in preparation for retirement? |
| Missing theoretical framework: You mention several theories throughout but never clearly state which one guides your study. Pick one model (Schlossberg's transition model would work well) and add a paragraph in your introduction explaining how it frames your research. | Thank you for your comment. I have now added a paragraph to the introduction detailing this integration. | L. 111-121  This research was guided by Schlossberg’s Transition Theory (Schlossberg, 2012), conceptualising transitions as events that require individuals to adapt to changes in roles, routines, relationships, and identities. Central to this model is the “4S system” (situation, self, support, and strategies), which proposes that an individual’s capacity to cope with transition is formed by personal characteristics, support system availability, and coping resources. Within professional sport, retirement symbolises a significant career and identity transition that may require structured support services to facilitate successful adjustment to post sporting life. Using this framework as a theoretical guide, the present study explores how existing support services within Irish horseracing contribute to the “support” dimension of the transition process, while also identifying perceived barriers and facilitators influencing jockeys’ engagement with these services as they prepare for retirement. |
| Create a table showing your 11 participants (their roles, how long they've been in position, whether they work directly with jockeys) | Many thanks for this comment. Upon reading the editors comments on ensuring anonymity and the small sample size, it is felt it is better not to include a table with further details on individual participants. |  |
| Explain your coding process more clearly - who did it? Was it just you or did others help? Did you check reliability? | Thank you for this comment regarding reliability. I have made it clearer in the manuscript around the reliability of the coding process and how the research team were actively involved. | L. 219-223  Codes were reviewed and discussed with members of the research team to support reflexive engagement with the data and to consider alternative interpretations. Themes were then reviewed, refined, named, and organised into themes and sub themes. |
| Your "rigor" section is only 4 lines - expand this to explain how you ensured quality (credibility, transferability, etc.) | Thank you for your comment. I have now expanded the rigor section. | L. 226-242  Peer debriefing (Janesick, 2007) was used to ensure quality standards throughout the research process. Peers (study supervisors) were given an opportunity to cast a critical eye over the project at various stages including development of the interview guide and write up of this paper. Consistent with reflexive thematic analysis, the researcher acknowledges their active role in generating meaning from the data. Familiarity with the horseracing context informed both engagement with participants and interpretation of their accounts. Reflexive practice was maintained throughout the analytic process, with ongoing consideration of how prior knowledge and assumptions shaped analytic decisions and theme development.  Given the small and interconnected nature of the Irish horseracing industry, potential power dynamics between the researcher and participants should be acknowledged. The researcher’s familiarity with the sector may have influenced how comfortable service providers felt discussing limitations in current support provision or barriers affecting jockeys’ engagement with services. Participants may have moderated their responses to protect organisational relationships or reputations within the industry. To reduce this influence, confidentiality and anonymity were emphasised, and participants were informed that the research was independent of governing bodies; however, this potential influence should be considered when interpreting the findings. |
| Organizational descriptions in wrong place: Lines 49-68 where you describe IHRB, HRI, Equipp, etc. - this belongs in your Methods section, not the Introduction. The Introduction should be about concepts and theory, not organizational structure. | Thank you for this comment. I have now added this information to the Participants section of the methods. | L. 143-155 |
| Discussion structure: Your discussion jumps around a bit and repeats things. Reorganize it with clear sections: summary of findings, what theories explain your results, comparison to other research, practical recommendations, limitations, future research, conclusion. | Many thanks for this comment | The discussion section has been shortened to make it more concise and impactful. |
| Weak jockey literature base: You only cite two studies on jockey retirement (both Australian, both from 2006-2007). That's okay - this literature simply doesn't exist yet! But acknowledge this explicitly and explain why it makes your study even more important as foundational work. | Many thanks for your comment. I have now added this explicitly to the introduction. | L.53-54  To the authors knowledge only one study on retirement in jockeys have been previously published (Speed, 2007). |
| Recommendations too vague: Instead of saying "stigma should be addressed," be specific. For example: "IHRB should require 10 hours of CPD annually for license renewal, including modules on career planning, financial literacy, and post-racing career options. Implementation: pilot program 2026, full rollout 2027. |  |  |
| A few typos and grammatical errors throughout (line 38: "themselves'", line 69: missing apostrophe, etc.) - needs a careful proofread | Many thanks for this comment. I have amended the points mentioned and a careful proofread has also been completed. |  |
| The philosophical section (lines 123-140) is quite long and uses jargon without defining it. Shorten to 4-5 sentences and explain what "emic epistemology" actually means for your study. | Thanks for this comment. I have shortened this section and made the explanation clearer. | L.124-134  This study was underpinned by ontological relativism and an emic epistemology. The experiences and meanings attributed by industry support service providers were interpreted as culturally situated within the Irish horse racing environment, rather than evaluated against external theoretical frameworks. Guided by Braun and Clarke’s (2019) Big Q paradigm, data was viewed as co-constructed through interaction between researcher and participant, with both positioned as “insiders” due to their shared experience of working with jockeys. The lead author’s prior engagement with the Irish jockey cohort enabled a reflexive use of subjectivity, enhancing interpretive depth, while maintaining methodological rigour (Silverman, 2000). Through semi-structured interviews, the study sought to generate an in-depth, contextually grounded understanding of perceived barriers and facilitators to support service utilisation, recognising that multiple, experience-dependent realities exist (Erlingsson & Brysiewicz, 2013). |

**Editor Comments (Purple highlights on manuscript)**

| Fundamental Sample Limitation  Critical Issue: The study only interviews service providers, not jockeys.  This is like evaluating a restaurant by asking only the chefs, never the customers  The title; An industry perspective is misleading—it should explicitly state service provider perspective | Thank you for this comment. I have changed the title to explicitly state that service providers are the study population.  Secondly, it has been added to the limitations section to discuss the lack of jockeys voices and to highlight that further research will next explore jockeys thought on this topic. | L.1-2  A service provider perspective in Irish horseracing on the availability of retirement specific support services for professional jockeys and perceived barriers and facilitators to their use.  L. 755-766  Several limitations should be considered when interpreting the findings of this research. First, this study reflects the perspectives of service providers and does not include the voices of current or retired jockeys. While participants offered insight into the availability a supports, their perspectives may not fully capture how these services are perceived or experienced by the jockeys themselves. Future research incorporating jockey perspectives would provide a more comprehensive understanding of service accessibility and engagement. |
| --- | --- | --- |
| The authors acknowledge this (lines 114-117, 767-770) but don’t adequately justify why they didn’t include both groups from the start  Impact on Validity: The findings represent assumptions rather than lived experiences. For  example, when discussing stigma toward help-seeking; we hear providers; interpretations, not  jockeys actual fears or concerns. | Thank you for this comment. The decision to interview service providers was intentional, as the study aimed to first examine the **structure, availability, and delivery of existing support services** and identify system-level barriers and facilitators. Service providers were best positioned to offer this broader organisational perspective.  We acknowledge that the perspectives of jockeys are essential to fully understand engagement with these services. This study is therefore intended as a **foundational step**, with future research planned to directly explore the **lived experiences and views of jockeys**, enabling a more comprehensive and athlete-informed understanding of career transition support.  In line with a similar comment from reviewer one, this has now been explicitly dealt with in both the introduction and limitations section. | L. 107-118  The aim of this study was to explore the perceptions of key stakeholders involved in the delivery of support services within Irish horseracing regarding the current provision of career and retirement support for jockeys. Specifically, the study sought to address the following research questions:  RQ1: What support services are currently available to assist Irish jockeys in preparing for retirement from professional horse racing?  RQ2: What barriers do stakeholders perceive as limiting Irish jockeys’ engagement with these support services?  RQ3: What facilitators do stakeholders perceive as promoting Irish jockeys’ engagement with support services in preparation for retirement?  Further research can then explore the thoughts of both current and retired jockeys on this topic, to gain an even deeper understanding and alternative perspective.  L.795-804  These should be explored further by gaining insight into the jockeys’ views on this topic. The research team have committed to carrying out this qualitative research and have the support of the IHRB to do so. Both current and retired jockeys will be interviews to explore their perspectives. Exemplar programmes to aid in career development and transitions used in other sports should be explored further to gain an in depth understanding of what they provide. This could then be used to guide the implementation of purpose specific multi-disciplinary support service for professional Irish jockeys in the future. The involvement of the IHRB in this research highlights the willingness on their behalf to implement recommendations made. Further research is needed to explore potential barriers that may arise on use of support services based on both jockey and trainer perspectives. |
| Methodological Weaknesses  Sample & Data Collection:  Only 11 participants for an entire industry seems insufficient.  Interview duration varies widely (16-48 minutes)—suggests inconsistent data depth  Inclusion criterion of 1 year in role is arbitrary and inadequately justified  Pilot interview conducted with someone who didn’t meet inclusion criteria (why?)  Analysis Rigor:  No evidence of inter-rater reliability or multiple coders  Only peer debriefing mentioned for rigor—insufficient for qualitative research  Unclear how many researchers were involved in coding  Risk of confirmation bias given all authors are insiders who have worked with jockeys for  years | Thank you for this comment. The structure of Irish horse racing means that there are only 11 organisations that can potentially provide support services to jockeys. Therefore each organisation was included with the representative involved/ most aware or support services being included in this current study.  The interview duration was unfortunately low in some instances due to a dearth of knowledge on the participants behalf with regard to support services availability/ provision.  Thank you for this comment. The one-year inclusion criterion was selected to ensure that participants had sufficient exposure to their role to provide **informed and reflective insights** into support service provision.  Thank you for this comment. I have now added a section in the methodology to explicitly state the researcher involved and the depth of their involvement.  Thank you for this comment. A similar comment was made by reviewer 1 and amendments to explain this dynamic have been added to the manuscript. | L.209-215 (blue highlight in manuscript)  Deductive methods were then applied used to organise data into themes and sub themes based on how the data codes related to the research questions (barriers and facilitators to support service use). Codes were reviewed and discussed with two members of the research team to support reflexive engagement with the data and to consider alternative interpretations. Themes were then reviewed, refined, named, and organised into themes and sub themes. A thematic map for each theme was then developed to help tell the story of the themes and to ensure a visible framework.  L.221-234 (Yellow highlight)  Consistent with reflexive thematic analysis, the researcher acknowledges their active role in generating meaning from the data. Familiarity with the horseracing context informed both engagement with participants and interpretation of their accounts. Reflexive practice was maintained throughout the analytic process, with ongoing consideration of how prior knowledge and assumptions shaped analytic decisions and theme development.  Given the small and interconnected nature of the Irish horseracing industry, potential power dynamics between the researcher and participants should be acknowledged. The researcher’s familiarity with the sector may have influenced how comfortable service providers felt discussing limitations in current support provision or barriers affecting jockeys’ engagement with services. Participants may have moderated their responses to protect organisational relationships or reputations within the industry. To reduce this influence, confidentiality and anonymity were emphasised, and participants were informed that the research was independent of governing bodies; however, this potential influence should be considered when interpreting the findings. |
| Insider Bias & Reflexivity  Concern: All authors have extensive history working in this industry.  While they acknowledge using a Big Q paradigm and insider status, there is minimal discussion of how they managed potential biases. No discussion of how their positions might have influenced what participants felt comfortable saying.  Given small community size, power dynamics between researchers and participants may have affected data  No reflexive journal mentioned | Thank you for this comment. A more extensive rigor section has now been added to address this potential bias.  Thank you for this comment. Whilst a formal reflexive journal was not maintained, reflexivity was ensured through continuous critical reflection and continuous note taking during and after interviews. This is reflected within the manuscript. The advice regarding using a formal reflexive journal will be taken onboard for future research endeavours. | L.226-234  Given the small and interconnected nature of the Irish horseracing industry, potential power dynamics between the researcher and participants should be acknowledged. The researcher’s familiarity with the sector may have influenced how comfortable service providers felt discussing limitations in current support provision or barriers affecting jockeys’ engagement with services. Participants may have moderated their responses to protect organisational relationships or reputations within the industry. To reduce this influence, confidentiality and anonymity were emphasised, and participants were informed that the research was independent of governing bodies; however, this potential influence should be considered when interpreting the findings.  L.202-216  To begin, the lead author became familiar with the data set by reading and re-reading the interview transcripts, notes were handwritten to ensure familiarisation. Notes were taken casually at this phase and there was no specific systematic approach, some notes were words and others were short sentences. Notes were then collated from each of the individual interviews to complete the familiarisation stage to capture the overall observation that might be useful for guiding coding. Inductive methods were used in the initial stages of data coding as the codes (barriers n = 24, facilitators n = 15) developed were taken directly from the raw data itself. Deductive methods were the applied used to organise data into themes and sub themes based on how the data codes related to the research questions (barriers and facilitators to support service use). Codes were reviewed and discussed with two members of the research team to support reflexive engagement with the data and to consider alternative interpretations. Themes were then reviewed, refined, named, and organised into themes and sub themes. A thematic map for each theme was then developed to help tell the story of the themes and to ensure a visible framework. Finally, this paper was written to report on the data collected within the results and discussion sections. |
| Overly Long, Repetitive Discussion  The discussion section (300+ lines) is approximately 30-40% too long:  Excessive repetition of findings  Some sections (e.g., mental health) tangentially related to core findings  Could be more focused and impactful if condensed  Theory is mentioned but not deeply integrated—feels added rather than foundational | Many thanks for this comment. On reflection I can see how the mental health section does not deepen the current studies findings. I have now removed it. This comment was also made by reviewer one and in light of these two suggestions this decision has been made.  Thank you for this comment. A section on theory has been added to the introduction section to ensure that the importance of the underlying guiding theory has been explicitly stated. | L. 96-106 (blue highlight)  This research was guided by Schlossberg’s Transition Theory (Schlossberg, 2012), conceptualising transitions as events that require individuals to adapt to changes in roles, routines, relationships, and identities. Central to this model is the “4S system” (situation, self, support, and strategies), which proposes that an individual’s capacity to cope with transition is formed by personal characteristics, support system availability, and coping resources. Within professional sport, retirement symbolises a significant career and identity transition that may require structured support services to facilitate successful adjustment to post sporting life. Using this framework as a theoretical guide, the present study explores how existing support services within Irish horseracing contribute to the “support” dimension of the transition process, while also identifying perceived barriers and facilitators influencing jockeys’ engagement with these services as they prepare for retirement. |
| Weak Practical Recommendations  While suggestions sound good (CPD, flexible education, career mapping), they lack depth:  Who pays? No discussion of funding or economic feasibility  How to change culture? Culture change is notoriously difficult; suggestions are  superficial  Multiple theories mentioned (Athletic Identity, Self-Determination Theory, Psychological  Safety) but loosely applied  No unified theoretical lens guiding interpretation  Theory feels sprinkled in rather than integral to analysis  Would benefit from consistent application of one or two frameworks | Thank you for your comment. We acknowledge that culture change is complex and often slow to implement. The intention of the discussion was to identify **initial, feasible facilitators** (accessible further education, normalising career planning and the development of an exemplar support programme) rather than to suggest that change can be achieved easily or in isolation. This is not seen as a ‘quick fix’ but rather a long term goal. The next stage of this research would be to investigate the jockeys perceptions so that co-ordinated action can then occur.  Information has now been added regarding the support from the Irish Horseracing Regulatory Board in relation to the research being conducted and it is hoped their support will lead to the feasibility of programme implementation in the near future.  Many thanks for this comment. As mentioned earlier the overarching theoretical framework has been discussed in the introduction. This theory also deal with identity as a cornerstone so it is therefore understandable to link in athletic identity theory. | L. 111-121  This research was guided by Schlossberg’s Transition Theory (Schlossberg, 2012), conceptualising transitions as events that require individuals to adapt to changes in roles, routines, relationships, and identities. Central to this model is the “4S system” (situation, self, support, and strategies), which proposes that an individual’s capacity to cope with transition is formed by personal characteristics, support system availability, and coping resources. Within professional sport, retirement symbolises a significant career and identity transition that may require structured support services to facilitate successful adjustment to post sporting life. Using this framework as a theoretical guide, the present study explores how existing support services within Irish horseracing contribute to the “support” dimension of the transition process, while also identifying perceived barriers and facilitators influencing jockeys’ engagement with these services as they prepare for retirement. |
| Ethics & Confidentiality  Irish horse racing is a small community—even anonymized, participants may be identifiable  Quotes are detailed enough that insiders could recognize speakers  No discussion of how this risk was managed or communicated to participants | Thank you for this comment. We acknowledge that Irish horseracing is a relatively small and close-knit community, which increases the risk of indirect identification even when standard anonymisation procedures are applied.  To address this, several steps were taken to protect participant confidentiality. Identifying details (e.g., specific roles, locations, or unique experiences) were removed or generalised during transcription and reporting. Careful consideration was also given to the selection and presentation of quotes to minimise the risk of recognition while preserving the meaning of participants’ accounts.  Participants were also made aware that each of the 11 organizations would be represented and so were aware of the small sample size of this research population.  Some quotes have been shortened to minimise recognisability. | L. 386-391 |
| Missing Critical Voices  Trainers: Portrayed as barriers to change, yet their perspectives are absent. This is problematic—you cannot fairly characterize a group without hearing from them.  Jockeys: As mentioned, their absence is the study is a fatal flaw. | Thank you for your comment. The absence of trainers and jockeys represents a limitation of the current study. The aim of this research, however, was to explore the issue from a **service provision and systems-level perspective,** and therefore participants were selected based on their roles within support service delivery.  We recognise that trainers are characterised in the findings as potential barriers, and we agree that their perspectives are essential to fully understand this dynamic. Similarly, the perspectives of jockeys are critical to capturing lived experience and engagement with support services. As such, we have revised the manuscript to more clearly acknowledge this limitation and to avoid overgeneralisation. | L.761-766  Several limitations should be considered when interpreting the findings of this research. First, this study reflects the perspectives of service providers and does not include the voices of trainers, current and retired jockeys. While participants offered insight into the availability of support services, their perspectives may not fully capture how these services are perceived or experienced by the jockeys themselves. Future research incorporating jockey perspectives would provide a more comprehensive understanding of service accessibility and engagement. |
| Presentation Issues  Some grammatical errors (though meaning is clear)  Inconsistent capitalization of stakeholder names (lines 58-61)  Formatting of subthemes inconsistent (lines 349, 367)  References occasionally missing (line 669—now corrected) | Many thanks for this comment. The manuscript has been reviewed and spell/ grammar checks have been conducted. |  |
| Generalizability  Findings are highly context-specific to Irish horse racing  Limited discussion of how cultural differences might affect transferability to UK,  Australia, or other racing jurisdictions.  The uniqueness of jockey culture is asserted but not compared systematically to other sports | Thank you for this comment. This study adopts a qualitative approach that emphasises **transferability.** We have strengthened the discussion to better articulate how the findings may be transferable to other contexts with similar characteristics, such as **high-performance sport environments with strong cultural norms, hierarchical structures, and early specialisation pathways.** | L. 759-764  The findings of this study are specific to Irish horseracing; however, they may be transferable to similar high-performance sport environments characterised by strong cultural norms, hierarchical relationships, and early specialisation. It is still advisable that differences in organisational structures and cultural contexts across jurisdictions and sports should be considered. |
|  |  |  |
|  |  |  |
|  |  |  |
|  |  |  |
